# Comprehensive Evaluation of Bacterial Blight Resistance and Gene Distribution in Common Wild Rice (*Oryza rufipogon*) from Hainan Province, China

**DOI:** 10.3390/plants15101492

**Published:** 2026-05-13

**Authors:** Xiaorong Xiao, Xiaowei Yan, Mengting Huang, Linan Zhai, Mingchao Zhao, Siyuan Huang, Bangji Zhou, Qingyu Wang, Huijian Wang, Yapeng Li, Yong Yun, Funeng Xing, Qingjie Tang

**Affiliations:** 1 Sanya Institute, Hainan Academy of Agricultural Sciences, Sanya 572000, China; xiaorong1990829@outlook.com (X.X.); yanxiaowei@hnaas.org.cn (X.Y.);; 2 Cereal Crops Institute, Hainan Academy of Agricultural Sciences, Haikou 571100, China; 3 State Key Laboratory of Tropical Crop Breeding, Sanya Institute of Breeding and Multiplication, Hainan University, Sanya 572000, China

**Keywords:** *Oryza rufipogon* Griff., bacterial leaf blight, phenotypic identification, resistance gene analysis

## Abstract

Bacterial blight (BB), caused by *Xanthomonas oryzae* pv. *Oryzae* (*Xoo*), is one of the most devastating diseases in rice worldwide. Common wild rice (*Oryza rufipogon* Griff.) inhabiting the high-temperature and high-humidity environments of Hainan Island has evolved strong disease resistance through natural selection, representing a valuable genetic reservoir for resistance breeding. However, large-scale characterization of resistance phenotypes, resistance genes, and their combinations remains limited. In this study, we evaluated BB resistance in 1511 Hainan common wild rice accessions against three *Xoo* strains (HNX004, PXO99^A^, and Z173) and analyzed the distribution of ten major known resistance genes (*Xa1*, *Xa3*, *Xa4*, *xa5*, *Xa7*, *Xa10*, *xa13*, *Xa21*, *Xa23*, and *Xa27*). Phenotypic evaluation revealed distinct strain-specific resistance patterns. Broad-spectrum resistance analysis (defined as moderate resistance or higher) revealed that 35 accessions (2.32%) were resistant to all strains, and 378 accessions (25.02%) showed resistance to two strains. Genotyping of known resistance genes revealed that, except for one accession, which lacked all tested genes but showed resistance to strain PXO99^A^, all other accessions carried every tested gene except *Xa21* and *xa13*. Interestingly, different *Xoo* strains exhibited distinct requirements for resistance genes, revealing a clear strain-specific resistance pattern. Notably, the number of resistance genes did not correlate with resistance level. Instead, specific complementary combinations, particularly *Xa1 + Xa10 + Xa23 + Xa4 + Xa7*, conferred the strongest broad-spectrum resistance. Our results demonstrate that gene quality and specific complementary combinations are more important than the absolute number of resistance genes. The identified resistant accessions and favorable gene combinations provide valuable resources for rice breeding programs.

## 1. Introduction

Rice (*Oryza sativa* L.), an important monocotyledonous plant, feeds over 50% of the world’s population and is cultivated in more than a hundred countries across the globe [1]. However, rice production has been threatened by a number of diseases. Bacterial blight (BB), caused by *Xanthomonas oryzae* pv. *oryzae* (*Xoo*), is one of the most destructive diseases and leads to substantial yield loss [2,3,4,5]. The high pathogenicity of BB is considered a major obstacle to achieving sustainable rice productivity, and controlling it is a cause receiving global attention. The most cost-effective method to control bacterial blight is to breed disease-resistant plants [4,6]. Therefore, the identification of resistance quantitative trait loci (QTLs) and genes from resistant germplasms is crucial for crop protection [7,8].

To date, about 49 BB genes have been identified in diverse rice sources [9,10,11]. It is worth noting that approximately 76% of these resistance genes are derived from cultivated rice. The rest come from wild rice. These include *Xa21*, *Xa23*, *Xa27*, *Xa29*, *Xa30(t)*, *Xa33*, *Xa35(t)*, *Xa38*, *xa45*, *Xa47*, *Xa48*, and *xa49(t)* [12,13,14,15,16,17]. Several R genes found in wild rice, like *Xa21*, *Xa23*, *Xa33*, and *Xa38*, have been widely employed in rice breeding programs [18,19]. Therefore, wild rice germplasm is a valuable genetic resource for improving rice resistance [20]. Identifying resistant varieties from wild germplasm has become a major research focus [21,22].

The genus *Oryza* contains 22 wild rice species, including *Oryza rufipogon*, *O. nivara*, *O. barthii*, *O. glumipatula*, *O. longistaminata*, *O. meridionalis*, *O. punctata*, *O. Minuta*, *O. officinalis*, *O. rhizomatis*, *O. eichingeri*, *O. alta*, *O. grandiglumis*, *O. latifolia*, *O. australiensis*, *O. coarctata*, *O. brachyantha*, *O. schlechteri*, *O. longiglumis*, *O. ridleyi*, *O. granulata*, and *O. meyeriana* [23,24,25]. Six of these have the AA genome. They are *O. rufipogon*, *O. nivara*, *O. barthii*, *O. glumipatula*, *O. longistaminata*, and *O. meridionalis.* This makes them more closely related to cultivated rice. Most notably, *O. rufipogon*, *O. nivara*, and *O. barthii* are primary gene sources. They can easily produce normal fertile progeny and provide transferable beneficial genes through free hybridization [26]. *O. rufipogon* (common wild rice, CWR) is the ancestor of Asian cultivated rice and the most important perennial germplasm resource for rice improvement [27,28,29]. Due to its unique growth environment, *O. rufipogon* has rich genetic diversity and high resistance to biotic and abiotic stresses. Thus, it has great potential for rice breeding and research [30,31]. *O. rufipogon* is widely distributed in tropical and subtropical regions of Asia. China represents the northernmost limit of its natural distribution [32,33,34]. *O. rufipogon* has been documented in eight provinces in southern China, namely Hainan, Guangxi, Guangdong, Yunnan, Hunan, Jiangxi, Fujian, and Taiwan [30]. Unfortunately, the populations in Taiwan disappeared in 1978 [35].

Hainan common wild rice grows at the lowest latitude in China. Like other common wild rice populations, it has evolved important resistance genes over time in a complex and changing environment. Despite this potential, several research gaps remain. Compared with other provinces, systematic evaluations of disease resistance in Hainan common wild rice remain relatively limited. Few studies have tested this germplasm against multiple *Xoo* races. As a result, the distribution of known resistance genes is unclear, and effective resistance gene combinations are still lacking. We hypothesize that Hainan common wild rice carries useful resistance genes. These genes can be linked to known markers. Some gene combinations may work better than single genes. To test this, we selected about 1511 common wild rice (*Oryza rufipogon*) germplasm resources collected from different counties and cities in Hainan Province, China. Their resistance to three *Xoo* strains (HNX004, PXO99^A^, and Z173) was tested to screen for resistant germplasm for use in breeding. Using ten known bacterial blight resistance genes (*Xa1*, *Xa3*, *Xa4*, *xa5*, *Xa7*, *Xa10*, *xa13*, *Xa21*, *Xa23*, and *Xa27*), we performed molecular identification on these tested materials. This revealed the existence and distribution of known resistance genes, as well as identified effective resistance gene combinations. Through association analysis of resistance genes and resistance phenotypes, we identified genes that contribute to resistance against individual *Xoo* strains and gene combinations that confer broad-spectrum resistance. The obtained data would provide a theoretical basis and technical reference for further mining and utilization of resistance genes in *O. rufipogon* from Hainan, as well as a material basis for cultivating rice varieties with disease resistance.

## 2. Results

### 2.1. Analysis of Resistance to Bacterial Leaf Blight in Hainan Common Wild Rice

#### 2.1.1. Resistance to Individual *Xoo* Strains

**Figure 1:**
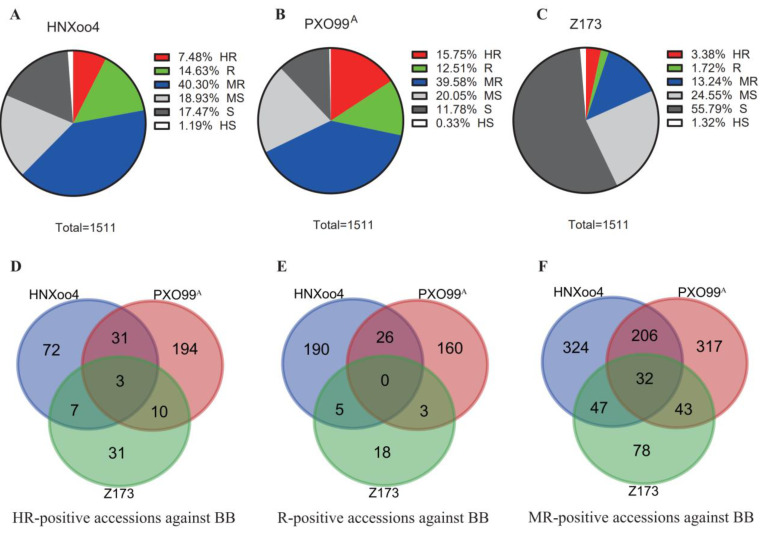
Percentage of resistance reaction and broad-spectrum resistance to three *Xanthomonas oryzae* pv. *oryzae* (*Xoo*) strains (HNXoo4, PXO99^A^, and Z173) in *Oryza rufipogon* from Hainan Province, China. (**A**–**C**) Pie charts showing the distribution of resistance level of *Oryza rufipogon* to three *Xoo* strains: (**A**) HNXoo4, (**B**) PXO99^A^, and (**C**) Z173. Resistance levels are classified as HR (high resistance), R (resistance), MR (moderate resistance), MS (moderately susceptible), S (susceptible), and HS (highly susceptible). Numbers in the legend indicate the percentage of each resistance level. The sum of percentages for each strain equals 100%. (**D**–**F**) Venn diagrams depict the number of unique and shared resistant accessions among the three *Xoo* strains, based on resistance classifications of (**D**) HR, (**E**) R, and (**F**) MR. BB: bacterial blight. Each circle represents one *Xoo* strain. The overlapping regions indicate accessions that are resistant to multiple strains. The central overlap in panel D identifies three accessions with broad-spectrum, strong resistance.

Approximately 1511 common wild rice germplasm resources were inoculated with three *Xoo* strains (HNXoo4, PXO99^A^, and Z173). The frequency distribution of resistance levels is shown in Figure 1A–C. The complete disease reaction heatmap is presented in Supplementary Figure S1. Of the plants evaluated, more than 62.4% of common wild rice exhibited resistance to HNXoo4 (HR = 7.48%; R = 14.63%; MR = 40.30%), while the rest of the common wild rice germplasm resources showed moderately susceptible to highly susceptible disease reaction (MS = 18.93%; S = 17.47%; HS = 1.19%) (Figure 1A). PXO99^A^ inoculation yielded a greater number of resistant plants than susceptible lines, with around 67.84% of the tested plants being resistant to PXO99^A^ (HR = 15.75%; R = 12.51%; MR = 39.58%), and 32.16% of plants exhibited susceptibility when inoculated with PXO99^A^ (MS = 20.05%; S = 11.78%; HS = 0.33%) (Figure 1B). Furthermore, the domestic standard pathogenic type IV strain Z173 was also used to test these 1511 accessions. As observed in Figure 1C, only 277 accessions (HR = 3.38%; R = 1.72%; MR = 13.24%) exhibited resistance to Z173, while the remaining 1234 accessions were susceptible, achieving scores of 5 to 9 (MS = 24.55%; S = 55.79%; HS = 1.32%).

#### 2.1.2. Broad-Spectrum Resistance Analysis

Based on the phenotype data of 1511 materials inoculated with three virulent strains of *Xoo*, 51 accessions showed high resistance to two or more virulent strains. Among them, only 3 accessions showed high resistance to all three virulent strains, 31 accessions exhibited high resistance to both the PXO99^A^ and HNXoo4 strains, 10 accessions exhibited high resistance to both the PXO99^A^ and Z173 strains, and 7 accessions exhibited high resistance to both the HNXoo4 and Z173 strains (Figure 1D). In addition, 26 accessions showed resistance to both the PXO99^A^ and HNXoo4 strains, 3 accessions showed resistance to both the PXO99^A^ and Z173 strains, and 5 accessions showed resistance to both the HNXoo4 and Z173 strains (Figure 1E). About 32 accessions showed moderate resistance to all three virulent strains, 206 accessions showed moderate resistance to both the PXO99^A^ and HNXoo4 strains, 43 accessions showed moderate resistance to both the PXO99^A^ and Z173 strains, and 47 accessions showed moderate resistance to both the HNXoo4 and Z173 strains (Figure 1F). Overall, the percentage of accessions resistant to all three strains was 2.32%, while for those resistant to two strains, it was 32.83%.

### 2.2. Genetic Diversity Analysis

The genetic diversity of Hainan common wild rice was characterized in our previous study using 32 SSR markers on 2038 accessions [36]. The 1511 accessions used in the current study are a subset of this larger collection. That study revealed high genetic variation, with Nei’s gene diversity (h) = 0.570, Shannon’s diversity index (*I*) = 0.975, and percentage of polymorphic loci = 96.27%. To directly assess whether any duplicate accessions exist within the subset of 1511, we performed pairwise Jaccard similarity analysis. The similarity values ranged from 0.1087 to 0.9780, with a mean of 0.3676 (SD = 0.0567). The distribution of pairwise similarities is shown in Supplementary Figure S2. Using the national standard threshold for duplicate identification (genetic similarity ≥ 99%; NY/T 1433-2014, GB/T 38551-2020), the results showed that no pair exceeded the threshold, with a maximum similarity of 0.9780 (well below 0.99).

### 2.3. Identification of Resistant Genes

#### 2.3.1. Distribution of Resistance Genes

**Figure 2:**
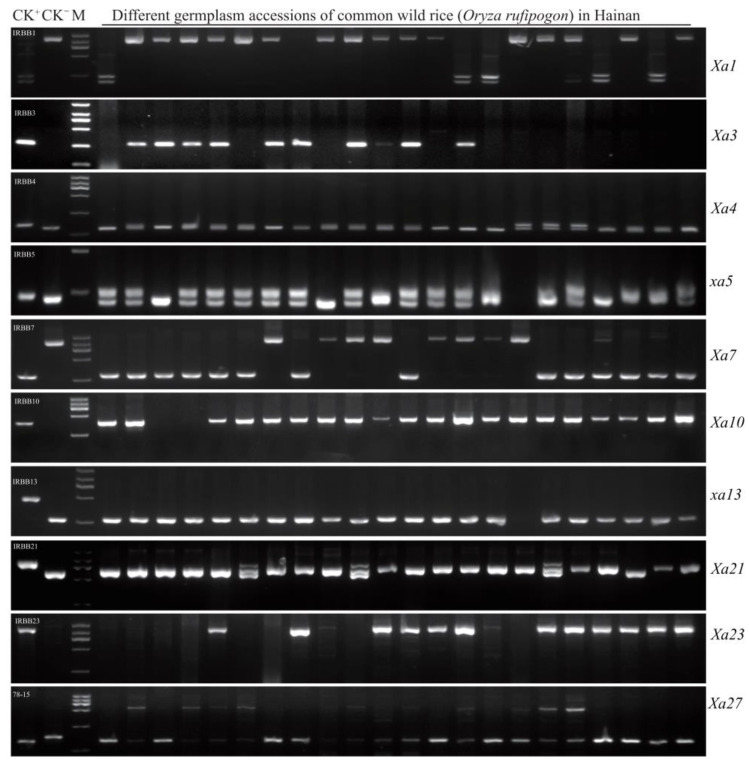
Representative gel electrophoresis of ten known *Xa* resistance genes (*Xa1*, *Xa3*, *Xa4*, *xa5*, *Xa7*, *Xa10*, *xa13*, *Xa21*, *Xa23*, and *Xa27*). M: DL2000 DNA marker; CK^+^: Positive control; IRBB: Near-isogenic lines (NILs) containing specific resistance genes; 78-15: The material contains the *Xa27* gene. CK^−^: Susceptible control line IR24; Lines 4–25: Different germplasm accessions of common wild rice (*Oryza rufipogon*) in Hainan. Accessions with band sizes matching the positive control (IRBB lines) were scored as “1” (gene present), those matching the negative control (IR24) as “0” (gene absent), and those with other band patterns as “2” (other allele). This figure illustrates the PCR-based genotype results used to determine the presence (1) or absence (0/2) of each resistance gene across the 1511 accessions.

**Figure 3:**
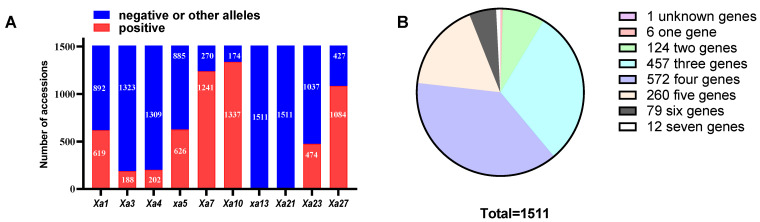
Number distribution and pyramiding analysis of bacterial blight resistance genes (*Xa1*, *Xa3*, *Xa4*, *xa5*, *Xa7*, *Xa10*, *xa13*, *Xa21*, *Xa23*, and *Xa27*) in 1511 accessions of *Oryza rufipogon* from Hainan Province, China. (**A**) Number distribution of ten tested *Xa* genes in 1511 accessions. Red bars indicate accessions with the gene (band matching positive control), and blue bars indicate accessions without the gene (band matching negative control or other patterns). Numbers on the bars represent the number of accessions: numbers on red bars indicate the count of accessions carrying the gene, and numbers on blue bars indicate the count of accessions not carrying the gene. (**B**) Pie chart of resistance gene count distribution. Each slice represents the percentage of accessions carrying a specific number of resistance genes. The numbers in the legend indicate the number of accessions, and the words in the legend indicate the number of resistance genes carried.

To identify BB genes, ten known resistance genes were used to identify the 1511 accessions. The distribution of resistance alleles differed substantially across the tested genes. The distribution of the ten known resistance genes among 1511 accessions is presented in Supplementary Figure S1. Representative PCR electrophoresis results of selected genes are shown in Figure 2. To validate the SSR-based genotype results, representative accessions carrying different resistance genes were selected for Sanger sequencing. The sequencing results confirmed the presence of the target resistance gene fragments and showed high consistency with the SSR marker data (Supplementary Figures S3–S12). These results demonstrate the reliability of the genotype data, which can be confidently used for subsequent statistical analyses. Based on the marker data (Figure 3A), the Xa1L primer (*Xa1* gene linked) showed that 40.97% (619 accessions) harbored the recessive resistance allele, while 59.03% (892 accessions) were negative or carried alternative alleles. A similarly intermediate frequency was observed for the *xa5* gene, where the linked RM122 primer identified 41.43% (626 accessions) carrying the resistance allele, with the remaining 58.57% (885 accessions) testing negative or carrying alternative alleles. Although also detected in a substantial portion of the panel, the prevalence of the *Xa23* allele (31%, 474 accessions) was slightly lower than that of *Xa1*. Nevertheless, the M5 primer (*Xa7* gene linked) identified 1241 accessions as carriers of the *Xa7* allele, but the remaining 270 accessions were negative or possessed other variants. Moreover, the *Xa10* allele exhibited a markedly high prevalence, present in 88.48% (1337 accessions) of the germplasm. Only 11.52% (174 accessions) lacked the dominant *Xa10* allele or possessed other variants. The distribution frequency of the *Xa27* allele closely paralleled that of *Xa10*. Consistent with *Xa7* and *Xa10*, *Xa27* (detected with marker M964) exhibited a high allele frequency in the germplasm. The resistant allele was present at a frequency of 71.74% (1084 accessions), whereas the susceptible or alternative alleles collectively accounted for the remaining 28.26% (427 accessions). In stark contrast, the MP1-2 primer (*Xa4* gene linked) identified 202 accessions harboring a resistant allele, while 1309 accessions were found to be negative or possess other alleles. The *Xa3* allele was also found at a low frequency, present in merely 188 (12.5%) of the 1511 accessions screened. Most notably, no accessions carrying the dominant resistance alleles for either *Xa21* (detected with primer pTA248) or *xa13* were identified across any of the 1511 accessions.

#### 2.3.2. Number of Resistance Genes per Accession

**Figure 4:**
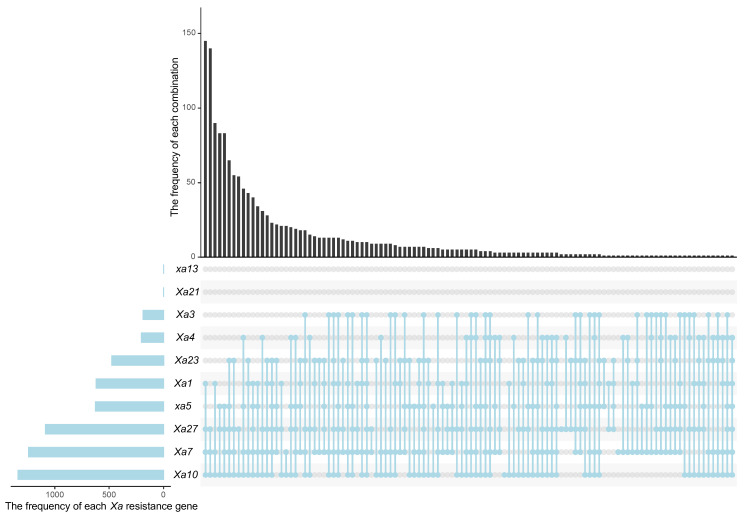
UpSet plot of *Xa* resistance genes in 1511 germplasm accessions. The UpSet plot visualizes the co-occurrence and distribution of the ten known *Xa* resistance genes *(Xa1*, *Xa3*, *Xa4*, *xa5*, *Xa7*, *Xa10*, *xa13*, *Xa21*, *Xa23*, and *Xa27*). The top bar chart illustrates the frequency of various *Xa* resistance gene combinations. The left bar chart represents the number of accessions containing each individual resistance gene. The bottom dot matrix delineates specific gene combinations, where connected black dots indicate the genes involved in each combination.

Notably, all accessions carried at least one of the known R genes, with the exception of a single accession in which none of the ten targeted genes were detected (Figure 3B). While only six accessions contained a single R gene, the majority (1504 accessions) harbored multiple genes in various combinations. The distribution was as follows: two genes (*n* = 124), three genes (*n* = 457), four genes (*n* = 572), five genes (*n* = 260), six genes (*n* = 79), and seven genes (*n* = 12). Interestingly, accession W787 displayed a resistant phenotype against PXO99^A^. However, none of the ten known R genes assayed were detected in this line, indicating that its resistance is likely conferred by an undiscovered genetic determinant. With the exception of two accessions (W787 and W1956), all 1509 materials harbored at least one of the genes *Xa7*, *Xa10*, or *Xa27*, where they occurred either singly or in combination with other genes (Figure 3B and Figure 4). The disease reaction scores and gene identifications for the tested accessions are summarized in Supplementary Figure S1.

#### 2.3.3. Gene–Strain Association Analysis

**Figure 5:**
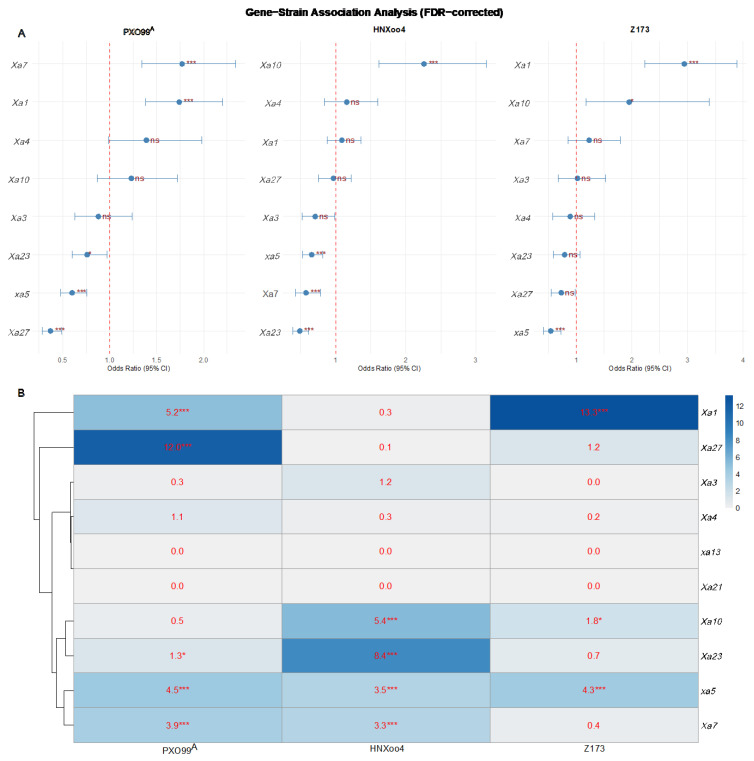
Gene–strain association analysis. (**A**) Forest plot of odds ratios (95% CI) for each gene–strain combination. The odds ratio (OR) quantifies the strength of association between individual resistance genes and bacterial blight resistance. Under the binarization scheme used in this study (resistant = 1; susceptible = 0; gene present = 1; gene absent = 0), OR > 1 indicates a positive association (the gene confers resistance to the corresponding strain), OR < 1 indicates a negative association (the gene is associated with susceptibility), and OR = 1 indicates no association. Horizontal lines represent the 95% confidence interval (CI). Red squares indicate significant associations (*p* < 0.05). Asterisks indicate FDR-adjusted significance levels (* *p* < 0.05, *** *p* < 0.001). ns indicates that the difference is not significant. (**B**) Heatmap of -log10(FDR-adjusted *p*-values) for each gene–strain combination. Colors range from light blue to dark blue, representing −log10(FDR-adjusted *p*-values) (darker colors indicate more significant associations). Numerical values in each cell are -log10(FDR-adjusted *p*-values), with attached asterisks indicating significance levels (* *p* < 0.05, *** *p* < 0.001). Rows are clustered based on association strength.

The association analysis revealed distinct resistance gene profiles for each *Xoo* strain. For strain PXO99^A^, five genes showed significant associations (−log10P > 1.3, *p* < 0.05): *Xa27* (−log10P = 12.0, OR (odds ratio) = 0.37), *Xa1* (−log10P = 5.2, OR = 1.74), *xa5* (−log10P = 4.5, OR = 0.6), *Xa7* (−log10P = 3.9, OR = 1.77), and *Xa23* (−log10P = 1.3, OR = 0.76). Among these, *Xa27* exhibited the strongest association with resistance to PXO99^A^. For strain *HNXoo4*, the most strongly associated gene was *Xa23* (−log10P = 8.4, OR = 0.49), followed by *Xa10* (−log10P = 5.4, OR = 2.26), *xa5* (−log10P = 3.5, OR = 0.66), and *Xa7* (−log10P = 3.3, OR = 0.58). *Xa3* exhibited borderline significance (−log10P = 1.2, *p* = 0.062). For strain Z173, three genes showed extremely strong associations: *Xa1* (−log10P = 13.3, OR = 2.94) and *xa5* (−log10P = 4.3, OR = 0.54). *Xa10* showed moderate association (−log10P = 1.8, *p* = 0.014). *Xa27* also exhibited borderline significance (−log10P = 1.2, *p* = 0.060). No other genes exhibited significant associations with this strain. Notably, *xa5* showed significant or marginal associations with all three tested strains (−log10P = 4.5, 3.5, and 4.3 for PXO99^A^, HNXoo4, and Z173, respectively), suggesting potential broad-spectrum resistance. *Xa1* was highly effective against PXO99^A^ and Z173 but not against HNXoo4. *Xa23* and *Xa7* were effective against PXO99^A^ and *HNXoo4* but showed no association with Z173. In contrast, *Xa4*, *xa13*, and *Xa21* showed no significant association with any of the three *Xoo* strains under the conditions of this study. A comprehensive summary of the association results is presented in Supplementary Table S1, and the association strength is visualized as a heatmap in Figure 5.

### 2.4. Gene Pyramiding and Statistical Analysis

#### 2.4.1. Frequency Distribution of Gene Combinations

**Figure 6:**
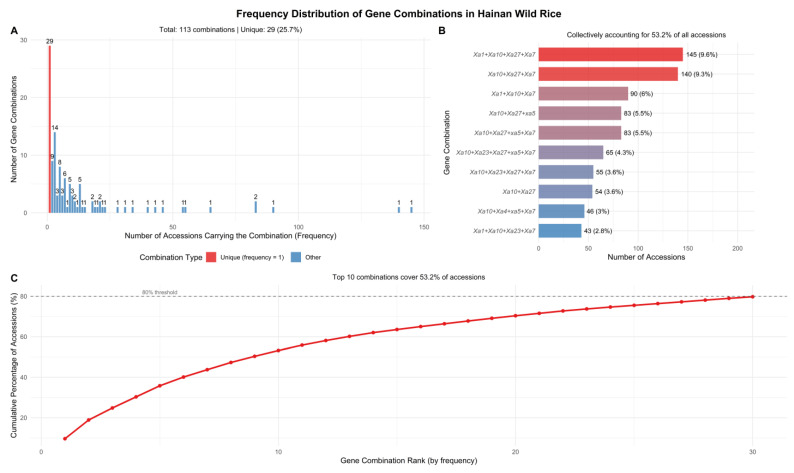
The frequency distribution of resistance gene combinations among 1511 Hainan common wild rice accessions. (**A**) A histogram of combination frequencies. Red bars represent unique combinations (frequency = 1) and blue bars represent non-unique combinations (frequency ≥ 2); numbers above bars indicate the count of combinations at each frequency level. (**B**) Top 10 most frequent gene combinations. The top 10 combinations collectively account for 53.2% of all accessions. Labels above bars show the frequency and percentage of total accessions. (**C**) A cumulative frequency distribution (Pareto chart) showing the cumulative percentage of accessions covered by combinations ranked by frequency. The red line represents the cumulative percentage, and the gray dashed line indicates the 80% threshold. Most combinations are rare or unique, reflecting the high genetic diversity of this wild rice collection.

A total of 113 distinct gene combinations were identified among the 1511 wild rice accessions. Approximately 25.7% of the gene combinations were unique, appearing in only a single accession (Figure 6A). The most prevalent combination was *Xa1 + Xa7 + Xa10 + Xa27*. The top ten most frequent gene combinations collectively accounted for 53.2% of all accessions (Figure 6B,C). The top 30 combinations were required to reach 80% cumulative coverage. This high proportion of low-frequency or unique combinations, together with the relatively gradual rise of the cumulative percentage curve, reflects the high genetic diversity of this wild rice germplasm.

#### 2.4.2. Correlation Between Resistance Gene Number and Disease Score

**Figure 7:**
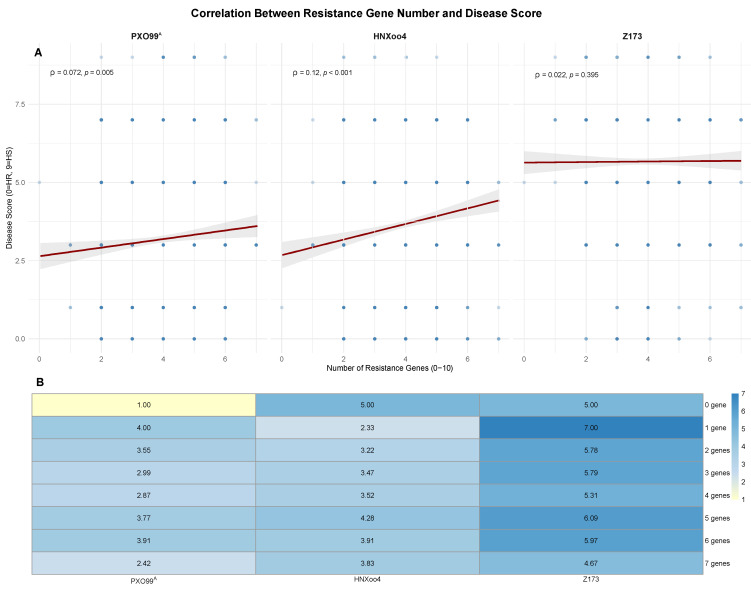
Correlation between resistance gene number and disease score. (**A**) Scatter plots showing the relationship between resistance gene number (0–10) and disease scores against three *Xoo* strains (PXO99^A^, HNXoo4, and Z173). Each point represents one accession. Spearman correlation coefficients (ρ) and *p*-values are shown in each panel. (**B**) Heatmap showing the average disease score for each gene count group against three *Xoo* strains (PXO99^A^, HNXoo4, and Z173). Gene count refers to the total number of functional resistance genes (out of the ten tested *Xa* genes) present in each accession. Accessions were grouped based on their gene count (0–7), and the mean disease score was calculated for each group. Color scale: lighter yellow (lower scores, more resistant) to blue (higher scores, more susceptible). Numbers within cells indicate mean disease scores for that specific gene count group and strain combination.

To determine whether carrying more resistance genes confers stronger resistance, we analyzed the correlation between the number of resistance genes per accession and the average disease score across three *Xoo* strains. Spearman rank correlation analysis revealed a statistically significant but extremely weak correlation between gene number and disease score (ρ = 0.12 for HNXoo4, ρ = 0.072 for PXO99^A^, ρ = 0.022 for Z173). The near-zero ρ value indicates that the relationship has no practical significance. Therefore, the absolute number of resistance genes does not meaningfully predict resistance level (Figure 7A). Heatmap analysis revealed distinct resistance patterns among the three *Xoo* strains (Figure 7B). Notably, Z173 was the most virulent strain among the tested accessions. Accessions with the same gene combinations showed higher disease scores against Z173 than against the other two strains. In contrast, PXO99^A^ was the most easily resisted strain. These results highlight that strain-specific resistance and gene quality are more important than the absolute number of resistance genes.

#### 2.4.3. Gene Combination Performance Against *Xoo* Strains

**Figure 8:**
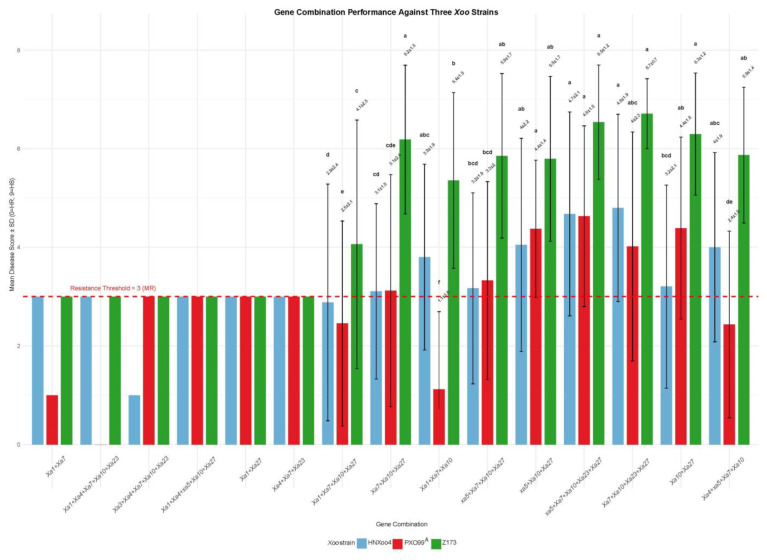
Performance of 15 gene combinations against three *Xoo* strains. Includes broad-spectrum (effective against all three strains) and high-frequency combinations (mean disease score ± SD) of three *Xoo* strains (PXO99^A^, HNXoo4, and Z173). Each gene combination is represented by three bars (blue for HNXoo4, red for PXO99^A^, and green for Z173). Numbers on bars indicate mean ± SD (only shown for combinations with frequency > 3). Different letters above bars indicate significant differences between gene combinations for each strain (Tukey HSD post hoc test, *p* < 0.05). The dashed red line indicates the resistance threshold (score ≤ 3). This figure facilitates the identification of gene combinations with broad-spectrum resistance (all three strains) or strain-specific resistance.

Given that gene number alone was not predictive of resistance, we next examined whether specific combinations of resistance genes are associated with enhanced resistance. The bar charts (Figure 8) revealed distinct resistance profiles among gene combinations against three *Xoo* strains. The results showed that resistance levels varied among different gene combinations. Broad-spectrum combinations, represented by *Xa1 + Xa10 + Xa23 + Xa4 + Xa7*, exhibited mean disease scores below three against all three strains, demonstrating stable resistance. In contrast, some combinations, such as *Xa1 + Xa10 + Xa27*, were effective against only one or two strains and showed weaker resistance to specific strains. The same gene combination showed differential resistance against the three *Xoo* strains, indicating strain-specific resistance patterns.

## 3. Discussion

### 3.1. Feasibility of Marker-Based Germplasm Screening

The leaf-clipping inoculation method has been widely employed for evaluating bacterial blight (BB) resistance in rice, providing reliable phenotypic data for resistance screening [37,38]. However, relying exclusively on phenotypic evaluation often limits the resolution needed to dissect the genetic basis of resistance, especially in genetically diverse germplasm such as wild rice. Molecular markers offer a powerful tool not only to accelerate the development of resistant varieties but also to precisely identify the presence of resistance genes in rice germplasm [39,40]. Previous studies have successfully utilized gene-linked markers to detect major BB resistance genes in both cultivated and wild rice materials. For instance, *xa13* and *Xa27* were detected in wild rice accessions [41], while *Xa4*, *xa5*, and *Xa21* were identified in Pakistani rice cultivars [42]. Another study further identified homologous fragments corresponding to *Xa1*, *Xa2/Xa31(t)*, *Xa14*, *Xa3/Xa26*, *Xa4*, *xa5*, *Xa10*, *Xa23*, and *xa25* in the surveyed Yunnan rice landraces [43]. These works demonstrate the applicability of marker-assisted selection across diverse Oryza populations and highlight the potential of wild rice as a valuable reservoir of resistance alleles.

### 3.2. Distribution of Resistance Genes in Hainan Wild Rice

In the present study, we extended this approach to a large panel of 1511 common wild rice (*Oryza rufipogon*) accessions from Hainan Province, a region known for its rich genetic diversity and unique ecological niche. By integrating phenotypic evaluation under artificial leaf-clipping inoculation at the mature stage with molecular genotyping using gene-specific markers, we systematically evaluated the distribution and combination of ten known BB resistance genes. Our results revealed that 99.93% of the accessions carried at least one of the tested resistance genes, with *Xa7*, *Xa10*, and *Xa27* being the most prevalent, along with other genes. In contrast, previous research has shown that the *Xa7* gene was present in only 4.91% of Chinese cultivated rice germplasm, while the *Xa23* gene was not detected at all [6]. Interestingly, we did not detect *xa13* or *Xa21* in any of the 1511 accessions examined in this study. Similarly, a study on Yunnan wild rice revealed that *xa5*, *xa13*, and *Xa21* were absent in all three wild rice species tested (*Oryza rufipogon*, *O. officinalis*, and *O. granulata*) [44]. The absence of *Xa21* in our collection can be explained by its evolutionary origin. *Xa21* was originally cloned from *Oryza longistaminata*, an African wild rice species. Notably, we identified one accession that exhibited resistance to PXO99^A^ but lacked all tested resistance alleles. This accession may possess novel resistance genes or alternative resistance mechanisms. Further validation through genetic mapping or functional studies is required. These findings support the strategy of leveraging wild germplasm to broaden the genetic base of BB resistance in cultivated rice and highlight the untapped genetic potential of Hainan common wild rice, consistent with earlier reports that wild rice species often harbor unique resistance alleles absent in cultivated varieties [38].

### 3.3. Strain-Specific Resistance Reflects Pathogen Diversity

The three *Xoo* strains used in this study showed different virulence patterns on Hainan wild rice accessions. Z173 was the most virulent. Only three genes (*Xa1*, *Xa5*, and *Xa10*) worked well against Z173. In contrast, more genes (*Xa1*, *xa5*, *Xa7*, *Xa23*, and *Xa27*) were effective against PXO99^A^, while the local strain, HNXoo4, was effectively resisted by *xa5*, *Xa7*, *Xa10*, and *Xa23*. This differential effectiveness reflects the presence or absence of specific TALEs in each strain, following the gene-for-gene paradigm. *Xa7*, *Xa23*, *Xa10*, and *Xa27* are executor R genes activated by cognate TALEs [45]. Their effectiveness against PXO99^A^ and HNXoo4 suggests that these strains carry the corresponding avirulence effectors, while Z173 likely lacks them. This finding is consistent with previous reports showing that PXO99^A^ carries the corresponding avirulence effectors (AvrXa7, AvrXa23, and AvrXa27), whereas in Z173, the sequences of avirulence genes such as *AvrXa7* and *AvrXa23* show divergence from those in virulent strains [46,47].

### 3.4. Gene Pyramiding Does Not Always Increase Resistance

Due to the continuous variation in *Xanthomonas oryzae* pv*. oryzae*, resistance conferred by the pyramiding of multiple genes is more stable and durable compared to that from a single gene [48]. Notably, in our study, 99.54% of the accessions harbored two or more resistance genes, suggesting that gene pyramiding may occur naturally in this wild population. Previous studies have found that the resistance conferred by the pyramiding of multiple genes is stronger than that from a single gene [49,50]. Likewise, we observed that accessions carrying at least two genes typically conferred higher resistance to strains PXO99^A^ and Z173 compared to those carrying only one resistance gene. However, a key finding of this study is that the number of resistance genes does not always correlate positively with resistance level. Spearman correlation analysis showed that the correlation coefficients were close to zero. We conclude that the absolute number of resistance genes does not meaningfully predict resistance level. This finding supports the notion that gene quality and specific complementary interactions, rather than the absolute number of pyramided genes, determine the effectiveness of resistance. This finding is consistent with previous studies showing that the *Xa1 + Xa10 + Xa23 + Xa4 + Xa7* combination confers strong and durable resistance in breeding programs. The limited avirulence gene repertoire of Z173 further supports this conclusion, as strains with fewer avirulence genes can evade recognition by a broader range of single R genes. Therefore, breeders should focus on selecting complementary gene combinations rather than simply adding more genes.

Although this study provides a valuable germplasm screening, the molecular mechanisms underlying the observed resistance remain to be elucidated. Other alleles were classified as gene-absent, but given that allelic variation within resistance genes can significantly affect resistance outcomes, we cannot rule out the possibility that these allelic variants may influence the phenotype. Future work should focus on the genetic mapping of resistance genes carried by these resistant accessions. Additionally, genome-wide association studies using high-density markers should be conducted to analyze other allelic variants of the ten known genes and to identify novel resistance loci beyond those tested here. Functional validation of these different allelic variants is also needed. These approaches will help translate our germplasm-level findings into actionable genomic insights for breeding. Field validation across multiple locations and seasons is required before practical application.

## 4. Materials and Methods

### 4.1. Materials

Plant material consisted of wild rice accessions and cultivated rice materials.

Wild rice accessions: The 1511 Hainan common wild rice accessions were collected from fourteen cities and counties in Hainan Province, China (Table 1). The plants were then planted at the research and experimental base, which is located in Chengmai at the Hainan Academy of Agricultural Sciences. A randomized complete block design (RCBD) was used with three replicates. In each replicate, three plants were grown per accession. Control materials were also planted in each replicate. Standard agronomic practices were applied. No chemical pesticides were used.

The rice materials, including nine bacterial blight-resistant *R* gene-containing monogenic lines (viz., IRBB1 (*Xa1*), IRBB3 (*Xa3*), IRBB4 (*Xa4*), IRBB5 (*xa5*), IRBB7 (*Xa7*), IRBB10 (*Xa10*), IRBB13 (*xa13*), IRBB21 (*Xa21*), and CBB23 (*Xa23*)), a cultivated rice parent (78-15) that harbors the *Xa27* gene, and one susceptible control material (JG30). These IRBB materials were provided by Professors Yinhua Chen and Zhihui Xia from Hainan University. These materials were originally collected from the Genetic Resource and Seed Division (GRSD) within the Bangladesh Rice Research Institute (BRRI), Gazipur, Bangladesh, and the International Rice Research Institute (IRRI), Philippines. 78-15 (contains *Xa27*) was kindly provided by Professor Yuehua Luo from Hainan University.

Bacterial blight strain: In this study, three *Xanthomonas oryzae* pv. *oryzae* (*Xoo*) strains were used: HNXoo4, PXO99^A^, and Z173. HNXoo4 is a local isolate obtained from diseased rice leaves collected in Hainan Province (laboratory isolate, unpublished). The inclusion of a local isolate allows assessment of resistance against regionally epidemic strains, which is directly relevant to breeding programs in Hainan. PXO99^A^ (Philippine race 6, corresponding to Chinese pathotype IX) is an international reference strain and represents the predominant pathotype in South China (56.57% of the *Xoo* population). Z173 (pathotype IV) is a dominant strain in the Yangtze River Basin, the largest rice-producing region in China. Together, these three strains cover geographic diversity (local, international, and national major rice regions) and virulence spectrum diversity, enabling identification of broad-spectrum resistance in wild rice germplasm.

### 4.2. Phenotypic Identification

#### 4.2.1. Pathogen Preparation

Before inoculation, the *Xoo* strains HNXoo4, PXO99^A^, and Z173 were inoculated on PSA medium (a combination of 10 g of peptone, 10 g of sucrose, and 1 g of L-glutamic acid, with water added to a total volume of 1000 mL and pH adjusted to 7.0~7.2 before adding 15 g of agar) and cultured at 28 °C for 3 d. Then, the bacterial cells were suspended with sterile distilled water to a density of 8 × 10^8^ cfu/mL (OD600 = 1).

#### 4.2.2. Inoculation Treatment and Disease Evaluations

All inoculations were performed during the same season. Temperature and humidity at the time of inoculation were recorded and maintained as consistently as possible. To ensure bacterial viability, the suspension was prepared fresh and used within 2 h. Inoculation was carried out using the clipping method [51]. Briefly, fully expanded leaves were selected, and the leaf tip (approximately 2.5–5.0 cm) was clipped with sterile scissors dipped in the bacterial suspension. Six fully expanded leaves were inoculated for each plant. Three replicate plants were used for each *Xoo* isolate. The rice variety JG30 was used as a susceptible control.

**Figure 9:**
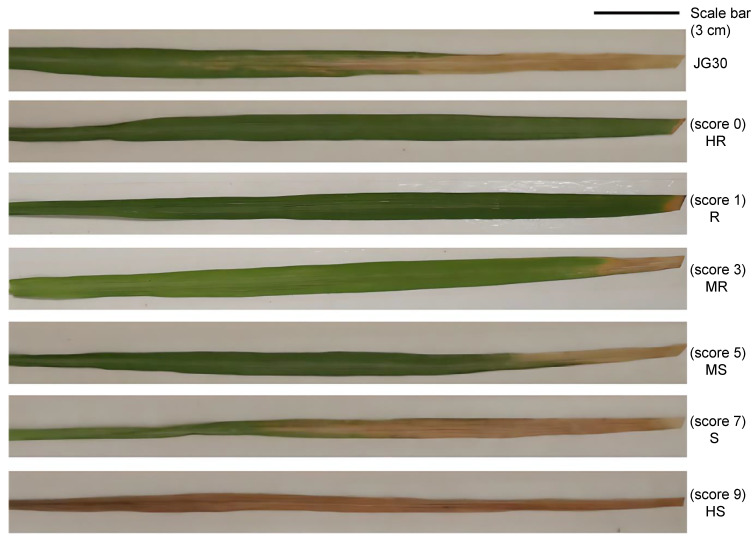
Different resistance grades based on the lesion length observed in wild rice leaves infected by *Xanthomonas oryzae* pv. *oryzae*. The leaf samples were harvested at 14 days post-inoculation (dpi) after leaf-clipping inoculation. The susceptible control variety Jingang 30 (JG30) is shown for comparison. Based on lesion length, disease reactions were classified into six resistance grades: HR (score of 0)—highly resistant, no visible lesion; R (score of 1)—resistant, lesion lengths of 0.1–1.0 cm; MR (score of 3)—moderately resistant, lesion lengths of 1.1–3.0 cm; MS (score of 5)—moderately susceptible, lesion lengths of 3.1–5.0 cm; S (score of 7)—susceptible, lesion lengths of 5.1–12.0 cm; HS (score 9)—highly susceptible, lesion length >12.0 cm. The scale bar = 3 cm.

The length of disease lesions on the inoculated leaves was evaluated 14 days after inoculation to assess the level and type of plant reactions. Lesion length was measured from the cut end at the leaf tip to the boundary between the healthy and diseased tissue. For each accession, 3–5 leaves were measured, and the mean value was calculated. Resistance levels were determined based on this mean value according to the criteria described in the Descriptors and Data Standard for Wild Rice Germplasm Resources [52]. Six classes of reactions were considered (Figure 9): a score of 0 (absence of symptoms) was considered as highly resistant (HR); a score of 1 (lesion lengths <1 cm) was considered as resistant (R); a score of 3 (lesion lengths of 1–3 cm) was considered as moderately resistant (MR); a score of 5 (lesion lengths of 3–5 cm) was considered as moderately susceptible (MS); a score of 7 (lesion lengths of 5–12 cm) was considered as susceptible (S); and a score of 9 (lesion lengths >12 cm) was considered as highly susceptible (HS). Plants with the first three types of reactions (score of 0, 1, or 3) were considered resistant, and the last three types (score of 5, 7, or 9) were considered susceptible to BB. These phenotype data were used to analyze the resistance genes involved in resistant and susceptible wild rice accessions.

### 4.3. Resistance Gene Analysis

Genomic DNA was extracted from young leaves of wild rice using the CTAB method. Ten diagnostic markers for known *Xa* genes, including *Xa1* (Xa1L), *Xa3* (Xa3/Xa26.R), *Xa4* (MP1-2), *xa5* (RM122), *Xa7* (M5), *Xa10* (Xa10.R), x*a13* (xa13), *Xa21* (pTA248), *Xa23* (Xa23), and *Xa27* (M964), were used to test the accessions resistant to BB (Table 2). Analysis of the reported disease resistance-related genes to rice bacterial leaf blight was performed by PCR.

PCR was performed in a 20 µL reaction mixture containing 5 µL of ddH_2_O, 1 μL of DNA template (2 ng·μL^−1^), 1 µL of each forward and reverse primer (10 μmol·L^−1^), and 12 µL of 2 × Es Tag Master Mix (Dye) (CWBIO, Taizhou, China). The amplification protocol comprised an initial denaturation at 95 °C for 3 min, followed by 30 cycles of denaturation at 95 °C for 30 s, annealing at 55–60 °C for 30 s, and extension at 72 °C for 45 s/kb, with a final extension step at 72 °C for 5 min. The PCR products were separated on 3% agarose gel by electrophoresis. The presence of resistance genes in wild rice was determined by comparing the fragments with corresponding control lines. Samples showing no or faint bands were subjected to three independent repetitions for final verification. A sample was scored as 1 (gene present) if the amplified band size matched that of the positive control, as 0 (gene absent) if it matched the negative control, and as 2 (other allele) if the band pattern differed from both controls. To confirm whether the amplified bands corresponded to the correct resistance genes, selected positive and negative amplification bands were subjected to Sanger sequencing. The obtained sequences were aligned with the reference sequences from control materials.

### 4.4. Association Analysis Between Resistance Genes and Xoo Strains

To evaluate the association between individual resistance genes and bacterial blight resistance, Fisher’s exact test was performed. Resistance phenotypes were binarized: HR, R, and MR = 1 (resistant); MS, S, and HS = 0 (susceptible). Genotypes were binarized: allele 1 = 1 (gene present); alleles 0 and 2 = 0 (gene absent). For each gene–strain combination, a 2 × 2 contingency table was constructed, and the odds ratio (OR) was calculated as OR = (a × d)/(b × c), where a = resistant accessions with the gene, b = susceptible with the gene, c = resistant without the gene, and d = susceptible without the gene. OR > 1 indicates stronger resistance, OR < 1 indicates weaker resistance, and OR = 1 indicates no association. The 95% confidence interval was also calculated. *p*-values were adjusted using the FDR method to control for multiple testing. Heatmaps were generated using −log10(P) values, with asterisks indicating significance levels (***, **, *).

### 4.5. Correlation Analysis Between Gene Number and Disease Score

To investigate the relationship between resistance gene number and resistance level, we calculated the number of resistance genes carried by each accession. The gene number was defined based on SSR marker results: allele 1 (present) was counted as 1, while alleles 2 and 0 (absent) were counted as 0. The gene number for each accession was the sum of scores across ten resistance genes (*Xa1*, *Xa3*, *Xa4*, *xa5*, *Xa7*, *Xa10*, *xa13*, *Xa21*, *Xa23*, and *Xa27*), ranging from 0 to 10. Resistance phenotypes were classified into six classes based on disease scores: highly resistant (HR = 0), resistant (R = 1), moderately resistant (MR = 3), moderately susceptible (MS = 5), susceptible (S = 7), and highly susceptible (HS = 9). The average disease score across the three *Xoo* strains (PXO99^A^, HNXoo4, Z173) was calculated for each accession. Spearman’s rank correlation analysis was performed to test the correlation between gene number and disease score for each strain, and the correlation coefficient (ρ) and *p*-value were calculated. One-way ANOVA was used to compare resistance differences among different gene count groups.

### 4.6. Gene Combination and Resistance Analysis

Based on SSR genotyping, resistance genes present in each accession (allele 1) were joined with “+” to form the gene combination name. Accessions with no detected genes were labeled as “None”. The frequency and mean disease score against three *Xoo* strains (PXO99^A^, HNXoo4, and Z173) were calculated for each combination. Broad-spectrum resistant combinations (mean score ≤3 against all three *Xoo* strains) were prioritized. Additional combinations were added by descending frequency to a total of 15. Mean disease score ± SD was calculated for each combination with frequency >3. One-way ANOVA followed by Tukey HSD post hoc test was performed for each strain to compare resistance levels among combinations (*p* < 0.05).

### 4.7. Data Visualization and Analysis

The Venn diagram was generated via an online website (http://www.biovenn.nl/, accessed on 30 January 2026). All statistical analyses and graphical visualizations (except Venn diagrams) were conducted using R software (version 4.5.2, R Foundation for Statistical Computing, Vienna, Austria).

## 5. Conclusions

In conclusion, our study provides a comprehensive genetic profile of BB resistance in a large collection of Hainan common wild rice. We confirm the utility of molecular markers for efficient gene detection and reveal substantial variation in resistance phenotype against three *Xoo* strains (PXO99^A^, HNXoo4, and Z173). Valuable broad-spectrum resistance resources were identified. *Xa7*, *Xa10*, and *Xa27* are the most prevalent genes, while *xa13* and *Xa21* were completely absent. The number of resistance genes does not always correlate with resistance level. Strain-specific resistance and gene quality are more important than the absolute number of resistance genes. The specific complementary combination *Xa1 + Xa10 + Xa23 + Xa4 + Xa7* provides the strongest and most stable broad-spectrum resistance. The resistant accessions and favorable gene combinations identified here represent valuable candidates for future marker-assisted breeding programs, pending functional and field validation.

In future work, we plan to integrate genotypic data from sequencing to enable allele-level analysis of known resistance genes. This will allow us to distinguish functional from non-functional variants and to assess haplotypic diversity within key loci. Furthermore, sequencing efforts will facilitate the mining of novel resistance genes, particularly in accessions that exhibit a resistance phenotype but lack known resistance gene fragments. Such an approach will deepen our understanding of the genetic architecture underlying bacterial blight resistance in these wild rice types and support the development of functional markers as well as durable resistant cultivars.

## Figures and Tables

**Table 1:** Number of common wild rice (*Oryza rufipogon*) accessions collected from different cities and counties in Hainan Province, China.

Origin (City/County)	Number of Accessions	Percentage (%)
Haikou	641	42.42
Lingao	9	0.60
Lingshui	10	0.66
Danzhou	22	1.46
Sanya	23	1.52
Qionghai	33	2.18
Ledong	37	2.45
Chengmai	54	3.57
Dongfang	95	6.29
Wanning	271	17.94
Wenchang	316	20.91
Total	1511	100

**Table 2:** Information about PCR primers used in the study.

Primer Names	Linked Xa Gene (s)	GenBank Accession No.	Primer Sequence (5′-3′)
Xa1L.F4	*Xa1*	AB002266.1	ATCAGGAACTTGAACTCCAG
Xa1L.R4			AACCACTGATTGCGGAAGG
Xa3/Xa26.RF	*Xa3*	DQ426645.1	GTCAGGTGGCATCCCTGCA
Xa3/Xa26.RR			AGGTGTTGGAGGATTGGCAT
MP1	*Xa4*	KU761305.1	ATCGATCGATCTTCACGAGG
MP2			TGCTATAAAAGGCATTCGGG
RM122F	*xa5*	AY643716.1	GAGTCGATGTAATGTCATCAGTGC
RM122R			GAAGGAGGTATCGCTTTGTTGGAC
M5F	*Xa7*	MW561276.1	CGATCTTACTGGCTCTGCAACTCTGT
M5R			GCATGTCTGTGTCGATTCGTCCGTACGA
Xa10.RF	*Xa10*	JX025645.1	ATGCAGCTGATGCTCACATTC
Xa10.RR			TCAGACGGGGGAAATCTCCT
xa13.F	*xa13*	DQ421394.1	GGCCATGGCTCAGTGTTTAT
xa13.R			AGCTCCAGCTCTCCAAATG
pTA248 F	*Xa21*	U37133.1	AGACGCGGAAGGGTGGTTCCCGGA
pTA248 R			AGACGCGGTAATCGAAAGATGAAA
Xa23.F	*Xa23*	KP123634.1	GCGGCATCACTAACATCA
Xa23.R			GAGGTAGGAGGAGGTAAGG
M964.F	*Xa27*	AY986492.1	TAGTGTCTAAATACAGGGACT
M964.R			GAGTACTTTGCTCTGATGCTC

## Data Availability

All data generated or analyzed during this study are included in this published article. Further enquiries can be directed to the corresponding author.

## Supplementary Materials

The following supporting information can be downloaded at https://www.mdpi.com/article/10.3390/plants15101492/s1, Figure S1: Disease reactions of 1511 *Oryza rufipogon* accessions from Hainan to three *Xanthomonas oryzae* pv. *oryzae* (*Xoo*) strains and their corresponding resistance gene profiles; Figure S2: Distribution of pairwise Jaccard similarity coefficients among 1511 *Oryza rufipogon* accessions from Hainan. Figures S3–S12: Nucleotide sequence alignment of ten resistance genes (*Xa1*, *Xa3*, *Xa4*, *xa5*, *Xa7*, *Xa10*, *xa13*, *Xa21*, *Xa23*, *Xa27*) between Hainan common wild rice accessions and controls. Table S1: Association analysis between resistance genes and *Xoo* strains.
