# Supplementary material for: Comprehensive Evaluation of Bacterial Blight Resistance and Gene Distribution in Common Wild Rice (Oryza rufipogon) from Hainan Province, China
Source: Plants (Basel). 2026 May 13;15(10):1492. doi: 10.3390/plants15101492 (PMC13210997; doi:10.3390/plants15101492)
Supplement: Supplementary file 1 [file plants-15-01492-s001.zip › plants-4236610-supplementary.docx]

**Figure S1.** Disease reactions of 1511 *Oryza rufipogon* accessions from Hainan to three *Xanthomonas oryzae* pv. *oryzae* (*Xoo*) strains and their corresponding resistance gene profiles. The figure is divided into two panels (A and B) arranged vertically. The columns (accessions) in both panels are in the same order, with each column representing one accession. (A) Disease reaction (DR) to three *Xoo* strains. PXO99^A^, HNXoo4 and Z173 represent three *Xoo* strains. Each row represents an *Xoo* strain. The color gradient from dark blue (Highly Resistant, HR) to dark red (Highly Susceptible, HS) represents the full spectrum of resistance levels: HR (0), R (1), MR (3), MS (5), S (7), and HS (9). (B) Distribution of ten known resistance genes. The ten resistance genes tested were *Xa1, Xa4, xa5, Xa7, Xa10, xa13, Xa3, Xa21, Xa23, and Xa27.* The presence (positive) and absence (negative) of genes were confirmed by SSR markers linked to the corresponding genes. Each row represents a resistance gene. Red indicates positive (1), blue indicates negative (0), and light yellow indicates alternative allele (2). Together, this integrated analysis reveals the relationship between specific resistance gene combinations and their corresponding disease reaction patterns across the three *Xoo* strains.

**Figure S2.** Distribution of pairwise Jaccard similarity coefficients among 1511 *Oryza rufipogon* accessions from Hainan. The histogram was generated based on SSR marker data from our collaborator‘s previous study (Zhai et al., 2024). In that study, 2,038 Hainan common wild rice accessions were genotyped using 32 SSR markers. The 1,511 accessions used in this study are a subset of this larger collection. Pairwise Jaccard similarity coefficients were calculated based on the 0/1 binary matrix derived from SSR marker data. The x-axis represents the Jaccard similarity coefficient (range 0-1, unitless), where values closer to 1 indicate higher genetic similarity. The y-axis represents the frequency (number of accession pairs). The red dashed line indicates the threshold for duplicate germplasm identification (≥ 99%) according to Chinese national standards (NY/T 1433-2014, GB/T 38551-2020).


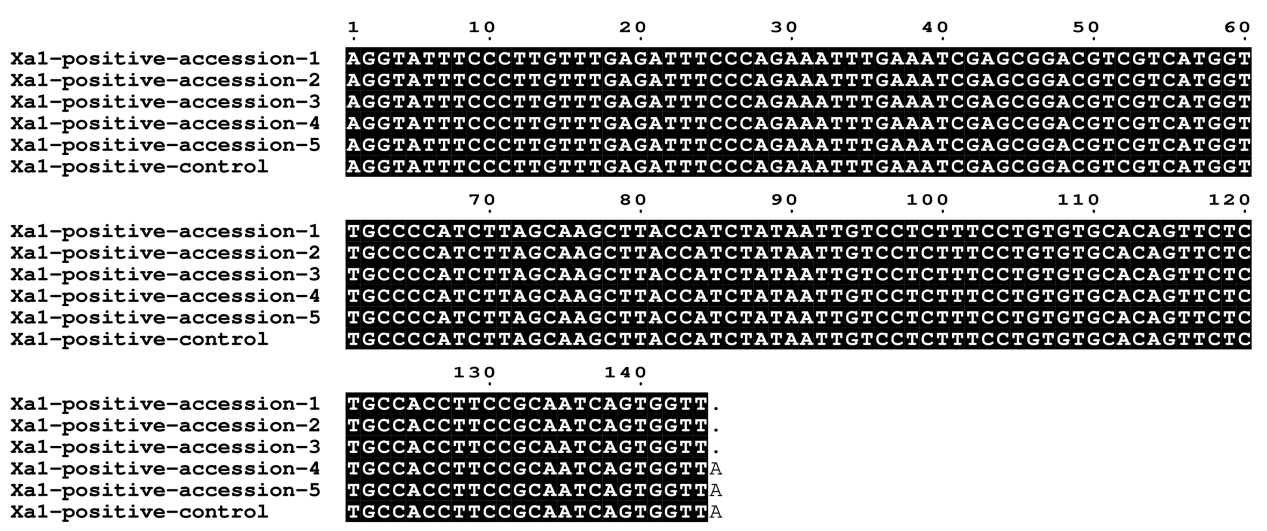


Figure S3. Nucleotide sequence alignment of *Xa1* resistance gene in *Oryza rufipogon* accessions and IRBB1. *Xa1* positive accession 1-5 represent the five selected wild rice germplasms that showed positive bands after PCR amplification, and *Xa1* positive control refers to the IRBB1.


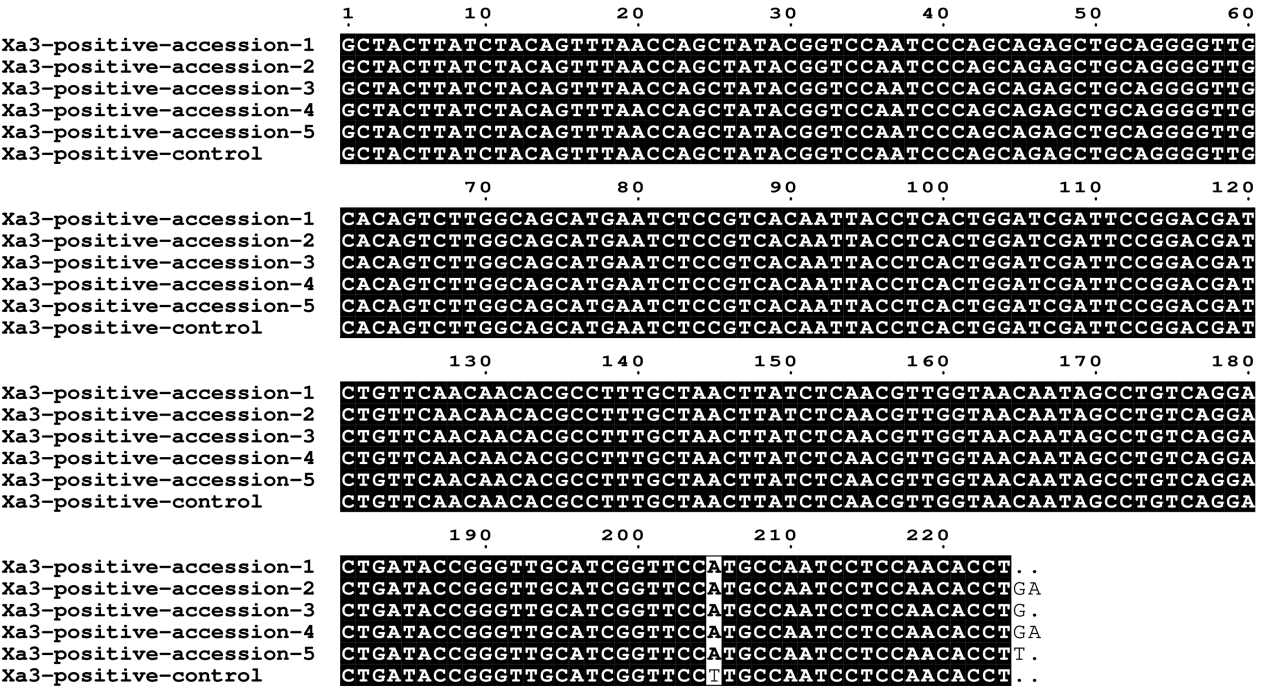


Figure S4. Nucleotide sequence alignment of *Xa3* resistance gene in *Oryza rufipogon* accessions and IRBB3. *Xa3* positive accession 1-5 represent the five selected wild rice germplasms that showed positive bands after PCR amplification, and *Xa3* positive control refers to the IRBB3.


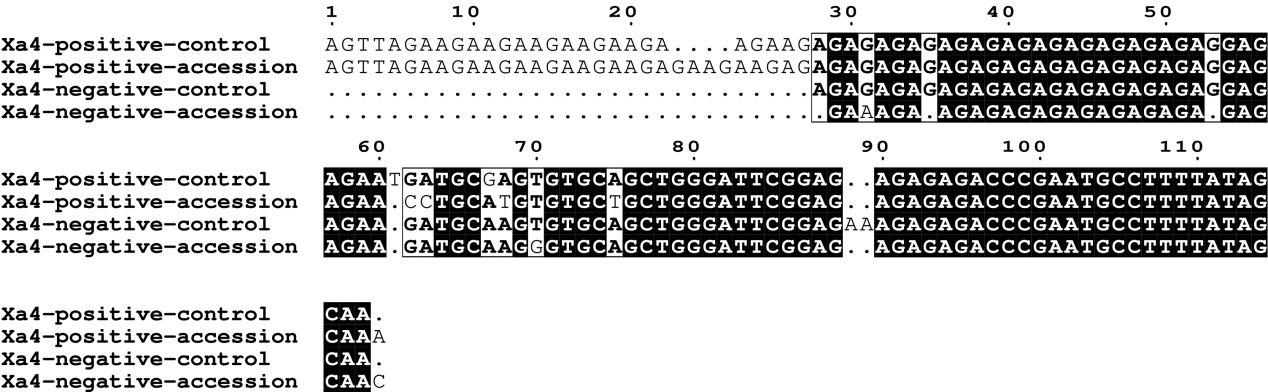
Figure S5. Nucleotide sequence alignment of *Xa4* resistance gene in *Oryza rufipogon* accessions, IRBB4 and IR24. *Xa4* positive accession represent the selected wild rice germplasm that showed positive bands after PCR amplification, and *Xa4* positive control refers to the IRBB4. *Xa4* negative accession represent the selected wild rice germplasm that showed negative bands after PCR amplification, and *Xa4* negative control refers to the IR24.


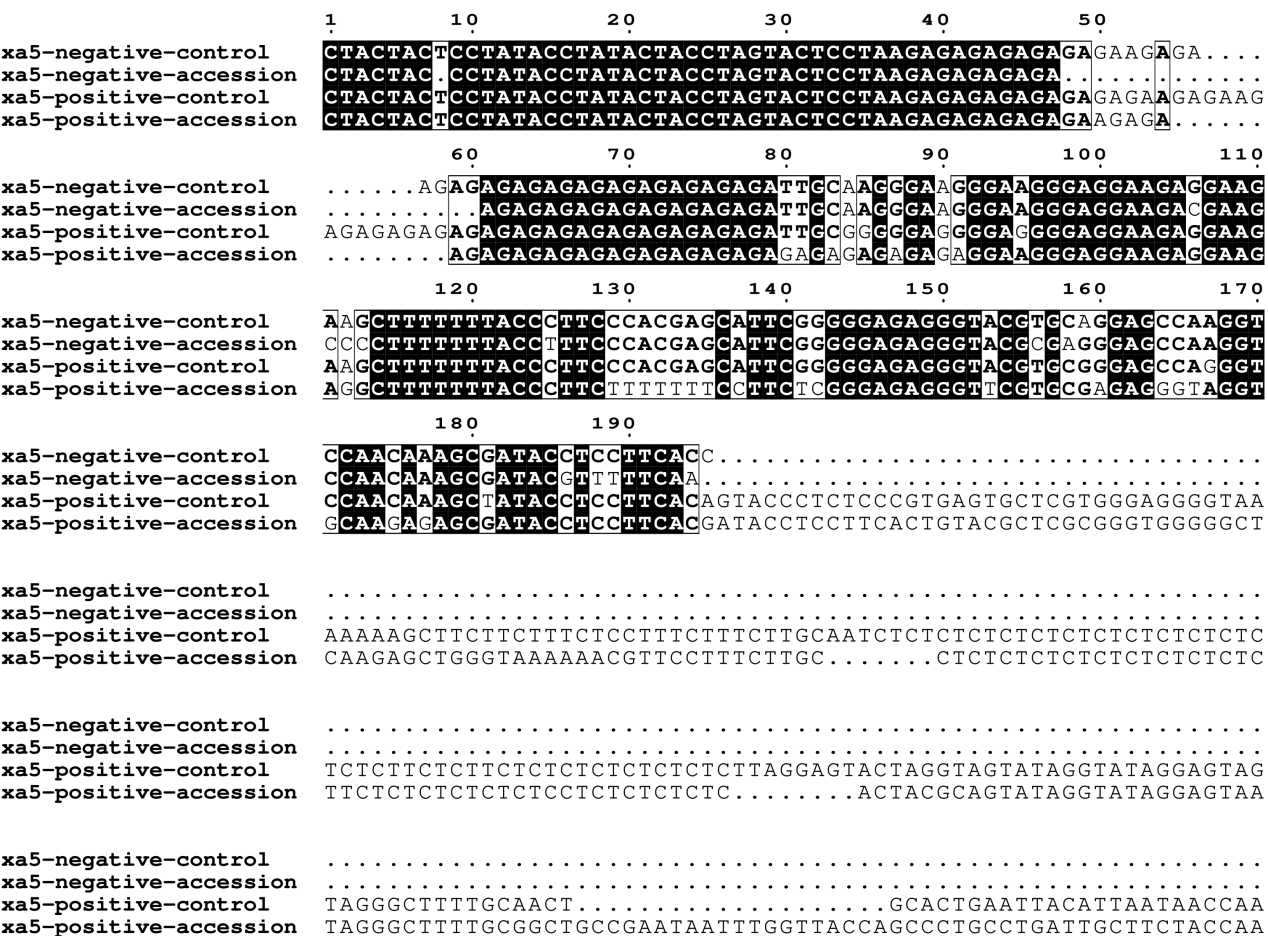


Figure S6. Nucleotide sequence alignment of *xa5* resistance gene in *Oryza rufipogon* accessions, IRBB5 and IR24. *xa5* positive accession represent the selected wild rice germplasm that showed positive bands after PCR amplification, and *xa5* positive control refers to the IRBB5. *xa5* negative accession represent the selected wild rice germplasm that showed negative bands after PCR amplification, and *xa5* negative control refers to the IR24.


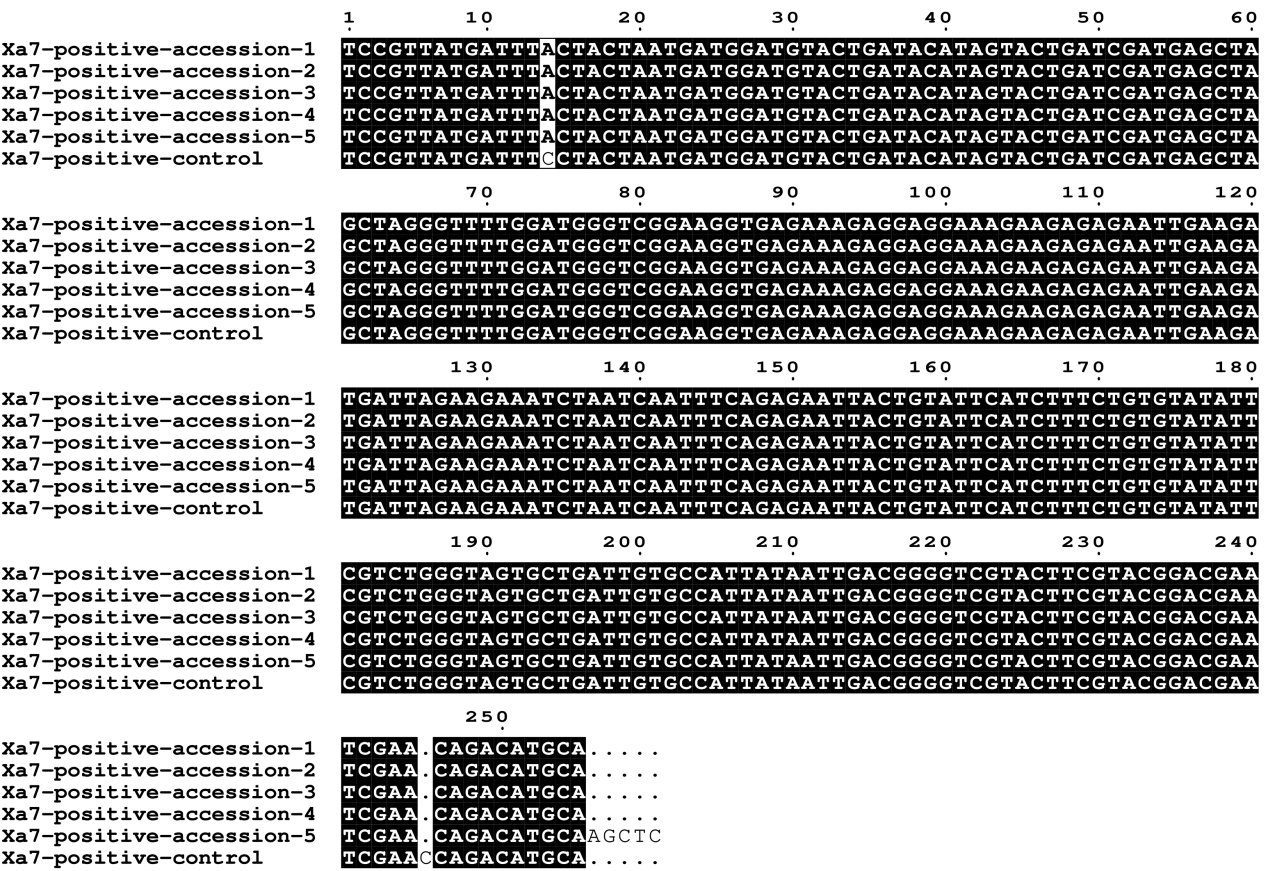


Figure S7. Nucleotide sequence alignment of *Xa7* resistance gene in *Oryza rufipogon* accessions from Hainan and IRBB7. *Xa7* positive accession 1-5 represent the five selected wild rice germplasms that showed positive bands after PCR amplification, and *Xa7* positive control refers to the IRBB7.


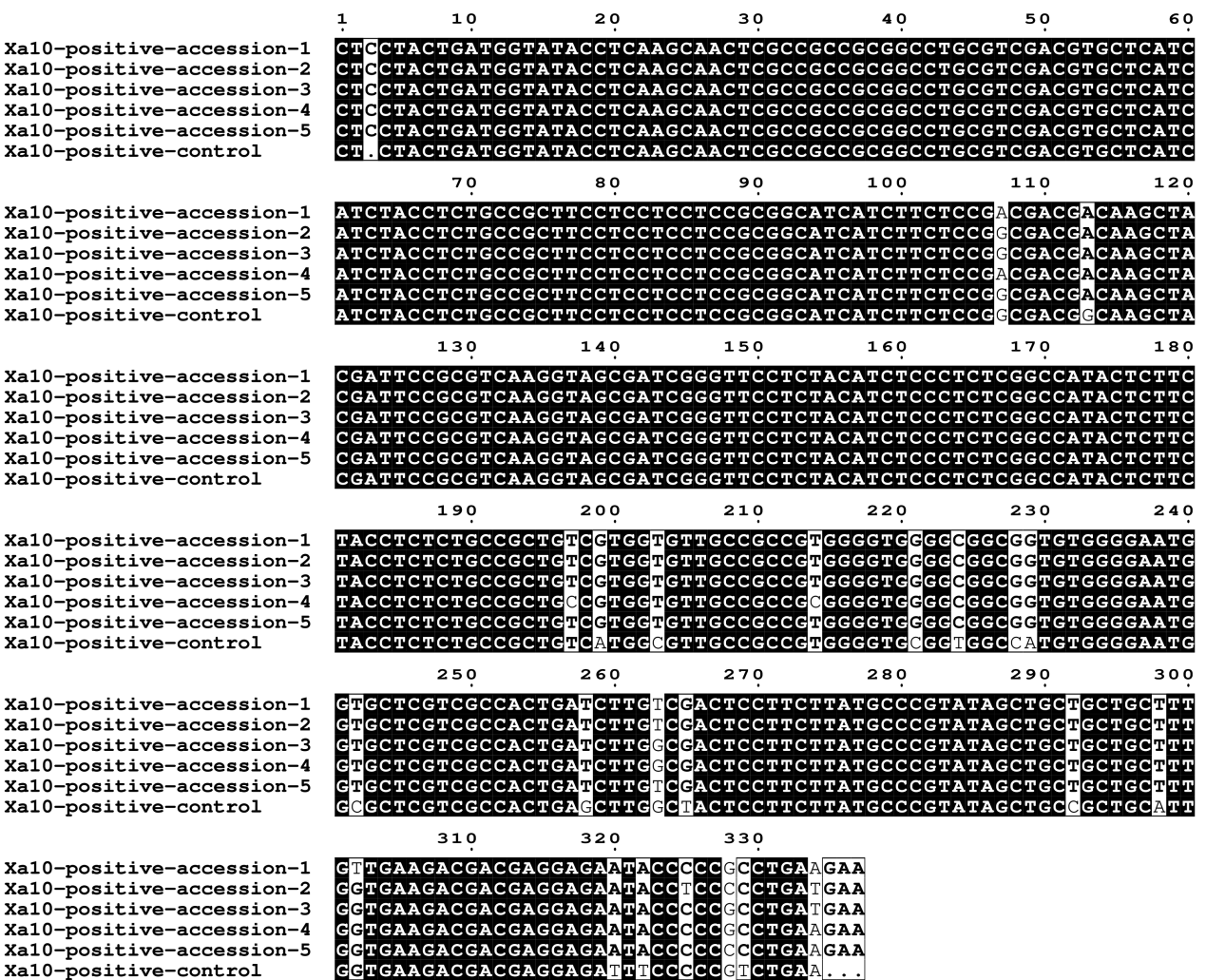


Figure S8. Nucleotide sequence alignment of *Xa10* resistance gene in *Oryza rufipogon* accessions from Hainan and IRBB10. *Xa10* positive accession 1-5 represent the five selected wild rice germplasms that showed positive bands after PCR amplification, and *Xa10* positive control refers to the IRBB10.


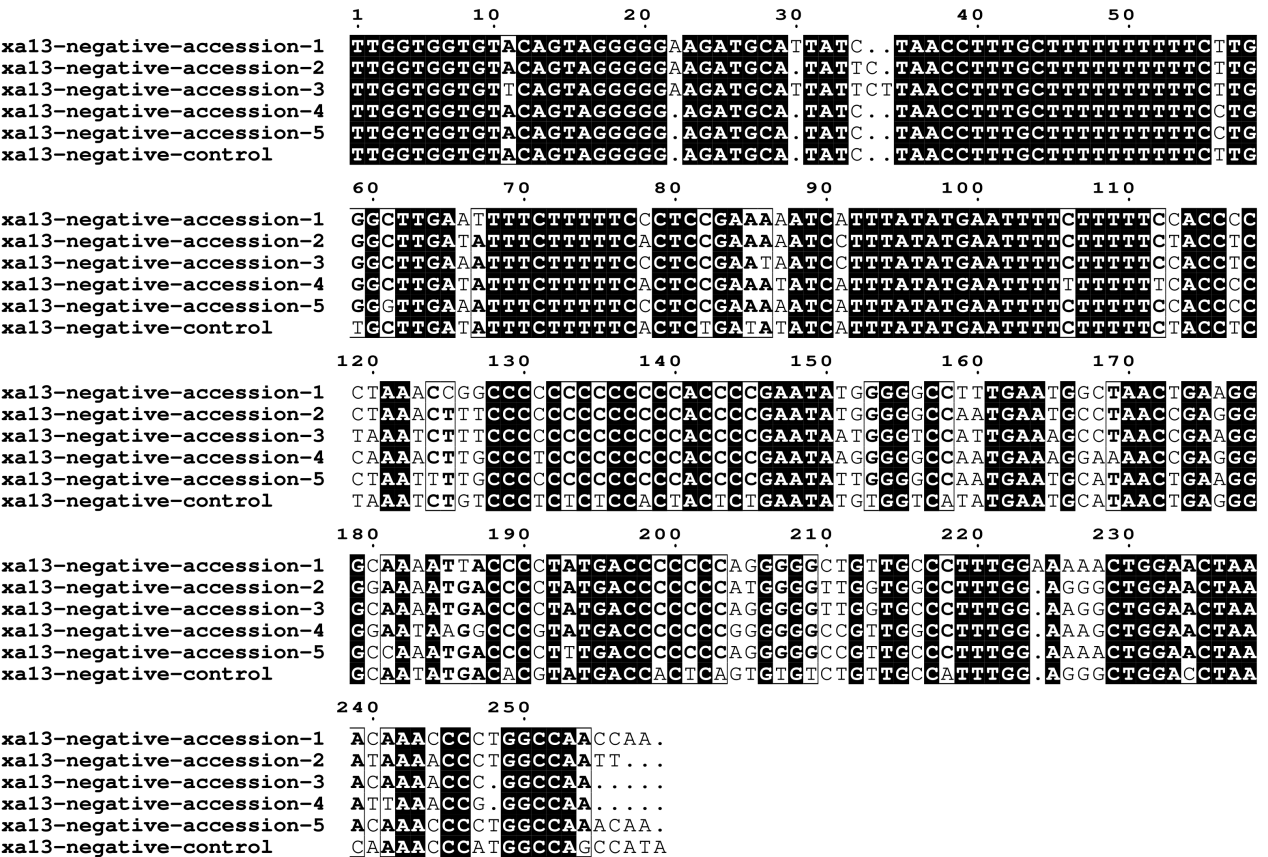


Figure S9. Nucleotide sequence alignment of *xa13* susceptible allele in *Oryza rufipogon* accessions from Hainan and IR24. *xa13* negative accession 1-5 represent the five selected wild rice germplasms that showed negative bands after PCR amplification, and *xa13* negative control refers to the IR24.


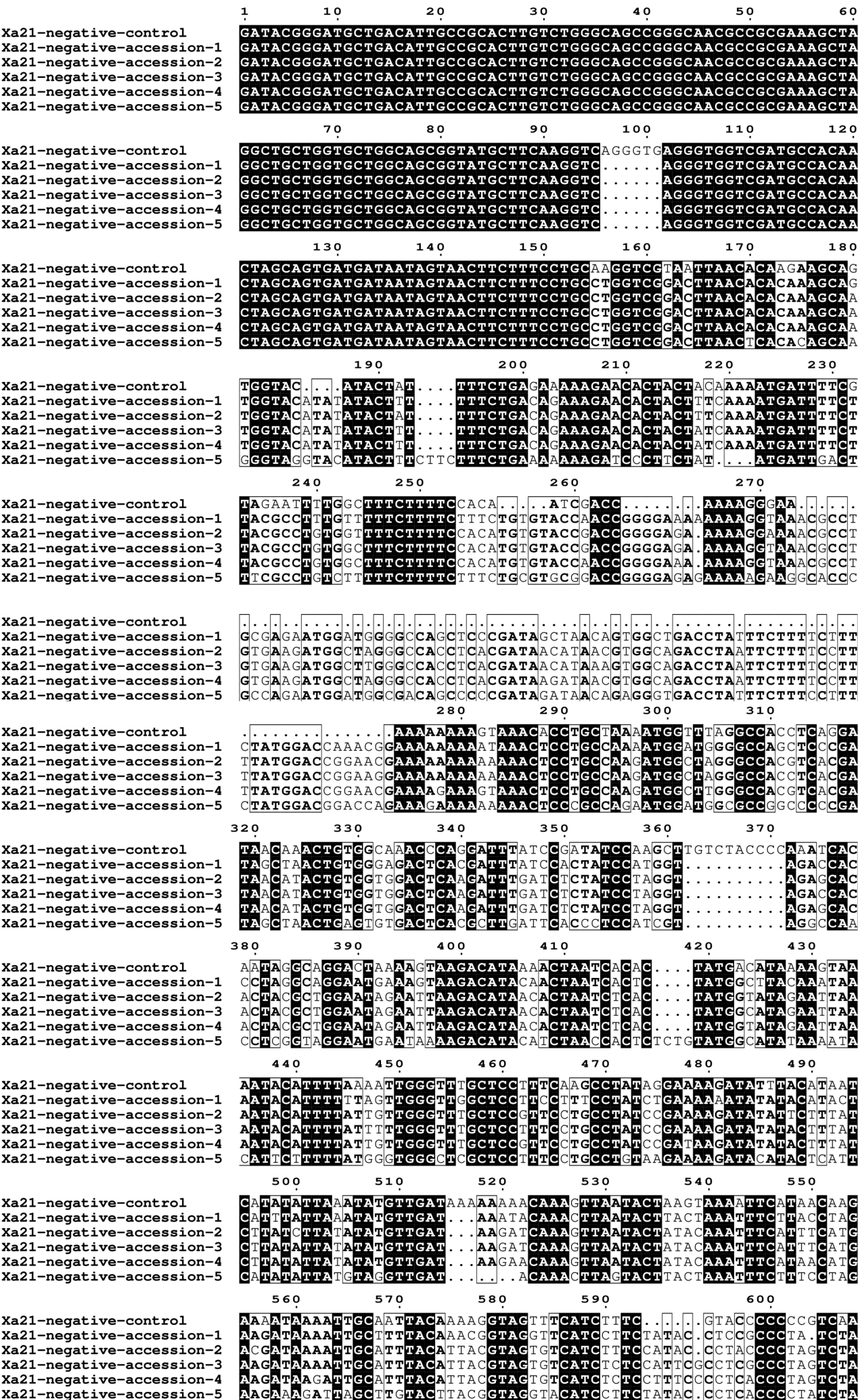


Figure S10. Nucleotide sequence alignment of *Xa21* susceptible allele in *Oryza rufipogon* accessions from Hainan and IR24. *Xa21* negative accession 1-5 represent the five selected wild rice germplasms that showed negative bands after PCR amplification, and *Xa21* negative control refers to the IR24.


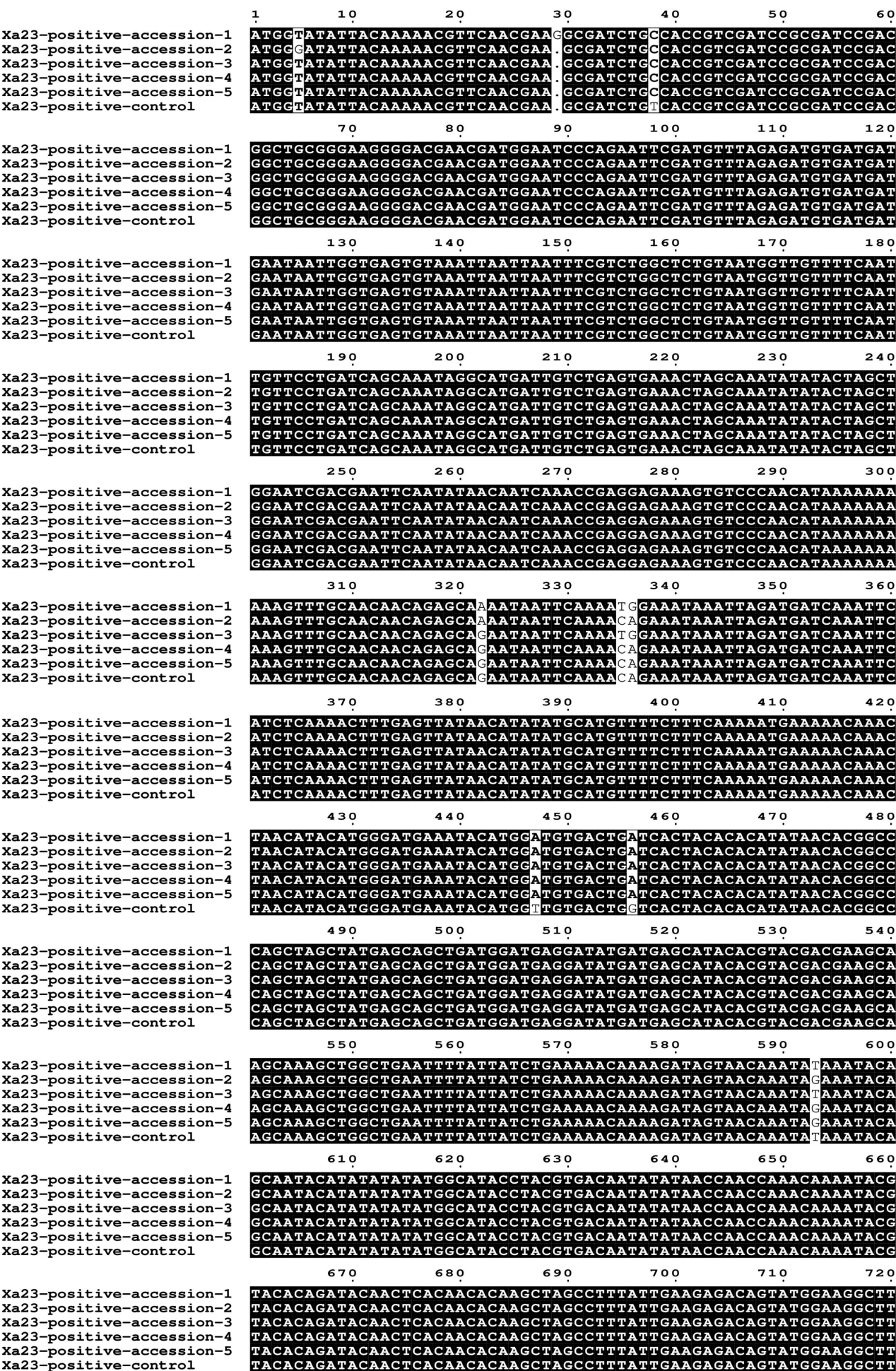


Figure S11. Representative sequence alignment of *Xa23* resistance gene in *Oryza rufipogon* accessions from Hainan and CBB23. *Xa23* positive accession 1-5 represent the five selected wild rice germplasms that showed positive bands after PCR amplification, and *Xa23* positive control refers to the CBB23.


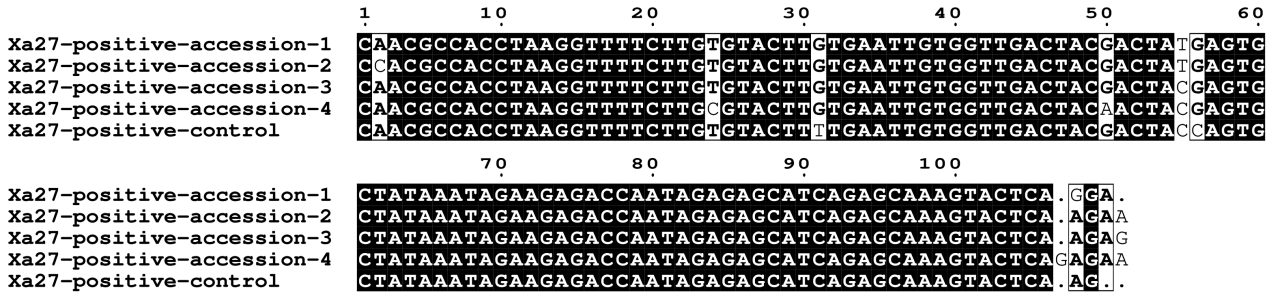


Figure S12. Nucleotide sequence alignment of *Xa27* resistance gene in *Oryza rufipogon* accessions from Hainan and control. *Xa27* positive accession 1-4 represent the four selected wild rice germplasms that showed positive bands after PCR amplification, and *Xa27* positive control represents the parent 78-15 containing *Xa27*.

Supplementary Table S1. Association analysis between resistance genes and *Xoo* strains.

| Strain | Gene | OR | CI_lower | CI_upper | P value | -log10 (P value) | Significant  mark |
| --- | --- | --- | --- | --- | --- | --- | --- |
| PXO99^A^ | *Xa1* | 1.74 | 1.38 | 2.2 | 6.72014969910524e-06 | 5.17262105242665 | *** |
| PXO99^A^ | *Xa3* | 0.88 | 0.63 | 1.24 | 0.518318850492874 | 0.285402996222853 | ns |
| PXO99^A^ | *Xa4* | 1.39 | 0.99 | 1.98 | 0.0841782009720288 | 1.07480036008324 | ns |
| PXO99^A^ | *xa5* | 0.6 | 0.48 | 0.76 | 2.98155946313364e-05 | 4.52555652482125 | *** |
| PXO99^A^ | *Xa7* | 1.77 | 1.34 | 2.34 | 0.000119451749340114 | 3.92280748573666 | *** |
| PXO99^A^ | *Xa10* | 1.23 | 0.87 | 1.72 | 0.321923855316816 | 0.492246839876707 | ns |
| PXO99^A^ | *Xa23* | 0.76 | 0.6 | 0.97 | 0.0480841197373406 | 1.31799833005387 | * |
| PXO99^A^ | *Xa27* | 0.37 | 0.28 | 0.49 | 1.0646329130491e-12 | 11.9728001117734 | *** |
| HNXoo4 | *Xa1* | 1.09 | 0.88 | 1.36 | 0.501875518773973 | 0.29940398845957 | ns |
| HNXoo4 | *Xa3* | 0.71 | 0.52 | 0.99 | 0.0622363187818781 | 1.20595610335766 | ns |
| HNXoo4 | *Xa4* | 1.16 | 0.84 | 1.6 | 0.493628729317615 | 0.306599572189928 | ns |
| HNXoo4 | *xa5* | 0.66 | 0.53 | 0.82 | 0.000344363550385462 | 3.46298282325845 | *** |
| HNXoo4 | *Xa7* | 0.58 | 0.43 | 0.79 | 0.000552581774547745 | 3.25760344324121 | *** |
| HNXoo4 | *Xa10* | 2.26 | 1.62 | 3.15 | 4.30777056665108e-06 | 5.36574743543228 | *** |
| HNXoo4 | *Xa23* | 0.49 | 0.39 | 0.62 | 4.39907538997569e-09 | 8.35663859515661 | *** |
| HNXoo4 | *Xa27* | 0.97 | 0.76 | 1.22 | 0.848884057997002 | 0.07115162237161 | ns |
| Z173 | *Xa1* | 2.94 | 2.23 | 3.89 | 4.52619113145719e-14 | 13.3442671106587 | *** |
| Z173 | *Xa3* | 1.02 | 0.67 | 1.53 | 0.919834826239502 | 0.0362901514511379 | ns |
| Z173 | *Xa4* | 0.89 | 0.58 | 1.33 | 0.682201663438867 | 0.166087225970193 | ns |
| Z173 | *xa5* | 0.54 | 0.41 | 0.73 | 5.16879853931841e-05 | 4.28661039469774 | *** |
| Z173 | *Xa7* | 1.23 | 0.85 | 1.79 | 0.396811027676185 | 0.401416266980645 | ns |
| Z173 | *Xa10* | 1.95 | 1.18 | 3.39 | 0.0142955082896503 | 1.84480039830069 | * |
| Z173 | *Xa23* | 0.79 | 0.59 | 1.07 | 0.198512025785845 | 0.702213178703213 | ns |
| Z173 | *Xa27* | 0.73 | 0.55 | 0.98 | 0.0596273557552002 | 1.22455444952376 | ns |

*Note: *, P < 0.05; **, P < 0.01; **, P < 0.001; OR, odds ratio.
